# Cyclic electron flow provides acclimatory plasticity for the photosynthetic machinery under various environmental conditions and developmental stages

**DOI:** 10.3389/fpls.2015.00800

**Published:** 2015-09-28

**Authors:** Marjaana Suorsa

**Affiliations:** Molecular Plant Biology, Department of Biochemistry, University of TurkuTurku, Finland

**Keywords:** acclimation, cyclic electron flow, development, electron transfer, environment, NDH complex, PGR5, PGRL1

## Abstract

Photosynthetic electron flow operates in two modes, linear and cyclic. In cyclic electron flow (CEF), electrons are recycled around photosystem I. As a result, a transthylakoid proton gradient (ΔpH) is generated, leading to the production of ATP without concomitant production of NADPH, thus increasing the ATP/NADPH ratio within the chloroplast. At least two routes for CEF exist: a PROTON GRADIENT REGULATION5–PGRL1–and a chloroplast NDH-like complex mediated pathway. This review focuses on recent findings concerning the characteristics of both CEF routes in higher plants, with special emphasis paid on the crucial role of CEF in under challenging environmental conditions and developmental stages.

## Introduction

Photosynthetic light reactions occur in four thylakoid membrane-embedded multiprotein complexes; photosystem (PS) II, cytochrome *b_6_f* (Cyt*b_6_f*), PSI and ATP synthase. In higher plants, these complexes are unevenly distributed along thylakoid membrane, as PSII complexes are mostly located in grana stacks, whilst PSI and the ATP synthase are enriched in the stroma-exposed thylakoids. The Cyt*b_6_f* complex has been traditionally assigned to be rather evenly distributed between the appressed and non-appressed regions, however, recent results from biochemical (Grieco et al., 2015) and immunolabeling (Armbruster et al., 2013) experiments suggest its predominant localization in the non-appressed thylakoid domains.

In linear electron flow (LEF), the PSs function in series and electrons are transferred all the way from water to NADP+ with concomitant production of NADPH and ATP. Cyclic electron flow (CEF), in contrast, recycles electrons around PSI by re-routing them from ferredoxin (Fd) to the plastoquinone (PQ). As a result, a transthylakoid proton gradient (ΔpH) is generated, leading to the production of only ATP. Consequently, CEF has been proposed to balance the ATP/NAPDH ratio. The ΔpH component forms part of the proton motive force (pmf), which drives the ATP synthase and can be monitored as electrochromic bandshift (ECS) from intact leaves. Besides ΔpH, also the transthylakoid electric potential (Δψ) contributes to pmf. The transthylakoid ΔpH is crucial for the regulation of LEF via two mechanisms: (i) increased ΔpH downregulates the Cyt*b_6_f* complex (“photosynthetic control”), which limits the electron flow from PSII toward PSI and thus protects PSI particularly upon sudden exposure to high light intensity (Joliot and Johnson, 2011; Suorsa et al., 2012). Furthermore, (ii) lumen acidification triggers induction of non-photochemical quenching (NPQ) of excess light energy, which protects PSII from photoinhibition (Li et al., 2009).

Two routes for CEF exist: (i) antimycin A sensitive pathway, which includes the PROTON GRADIENT REGULATION5 (PGR5) and PGR5-LIKE PROTEIN1 (PGRL1) proteins (Munekage et al., 2002, 2004; DalCorso et al., 2008) and (ii) antimycin A insensitive pathway, which transfers electrons via the NAD(P)H dehydrogenase-like (NDH) complex (Ifuku et al., 2011) [for a recent reviews, see (Leister and Shikanai, 2013; Shikanai, 2014]. Both of these pathways receive electrons from Fd (Yamamoto et al., 2011; Hertle et al., 2013). It has been estimated that in C3 plants, roughly one tenth of the total electron flow derives from CEF (Avenson et al., 2005). However, it is likely that under some specific conditions, such as upon onset on light illumination and under certain environmental and developmental conditions (see below), the proportion of CEF might be substantially higher. Based on the current knowledge, the major part of CEF in C3 plants is thought to be driven by the PGR5–PGRL1 pathway (Avenson et al., 2005; Wang et al., 2014). Besides defective pmf, the *pgr5* mutants of both *Arabidopsis* and rice have also been shown to exhibit an elevated proton conductance of the ATP synthase (Avenson et al., 2005; Nishikawa et al., 2012; Wang et al., 2014), as well as an increased amount of the ATP synthase β subunit (Suorsa et al., 2012). In contrast, the *ndh* mutants show an ATP synthase activity similar to that of wild type (WT; Wang et al., 2014). Whilst a deficiency of either of CEF pathways does not lead to a visual phenotype under standard growth conditions, a complete inhibition of CEF in the *Arabidopsis pgr5 crr-2* double mutant deficient in both pathways severely impairs plant growth and performance (Munekage et al., 2004), implying that CEF is essential for photosynthesis even in the C3 species.

This review focuses on the recent findings concerning the characteristics of both CEF routes, with special emphasis paid on the crucial role of CEF in higher plants under challenging environmental and developmental conditions.

## The PGR5–PGRL1 Route of CEF

The crucial role of the PGR5 protein for the maintenance of proper ΔpH was demonstrated already more than 10 years ago (Munekage et al., 2002) and a few years later, its interaction with PGRL1 was characterized (DalCorso et al., 2008). Whilst PGR5 does not contain a transmembrane helix, it is present in purified thylakoid fractions (Munekage et al., 2002). The interaction of PGR5 with the membrane-spanning PGRL1 likely takes place via conserved Cys residues present in both PGR5 and PGRL1 (Hertle et al., 2013). *In vitro* –assays indicate that PGRL1 interacts also with Cyt*b6f*, Fd, the PSI subunit PsaD, and with both isoforms of the Fd-NADP^+^-oxidoreductase (FNR) (DalCorso et al., 2008). Furthermore, it was recently demonstrated that the PGRL1-PGR5 complex is capable of accepting electrons from Fd, and PGRL1 can reduce quinones, indicating that the long-sought hypothetical ferredoxin-plastoquinone reductase (FQR) has finally been experimentally characterized (Hertle et al., 2013).

PROTON GRADIENT REGULATION5 is present in all photosynthetic organisms, whereas PGRL1 is specific for green algae and plants (DalCorso et al., 2008). In the green algae *Chlamydomonas reinhardtii* (*Chlamydomonas*), PGRL1 has been shown to be present in the protein complex mediating CEF under state 2 conditions, together with PSI, its light harvesting complex (LHCI), LHCII, Cyt*b_6_f*, and FNR (Iwai et al., 2010). PGR5, in contrast, was not found to be part of the complex (Iwai et al., 2010). However, the recently characterized *Chlamydomonas pgr5* and *pgrl1* mutants (Petroutsos et al., 2009; Tolleter et al., 2011; Dang et al., 2014; Johnson et al., 2014) exhibit characteristics resembling *Arabidopsis pgr5* and *pgrl1* mutants, indicating that also the PGR5 protein of *Chlamydomonas* has a role in CEF.

In *Arabidopsis*, PGRL1 is encoded by two genes, the PGRL1A isoform (encoded by the *AT4G22890* gene) of which has been shown to be phosphorylated by the STN8 kinase (Reiland et al., 2011). Even though the *stn8* mutant was capable of reaching similar overall rate of CEF as compared to WT, its capacity to maintain CEF upon shift from darkness to light was lowered (Reiland et al., 2011), indicating that the STN8 kinase regulates the kinetics of CEF. However, as not only green algae, diatoms, mosses, and lycophytes, but also the monocot species lack the phosphorylated threonine from their PGRL1A sequence, this regulatory mechanism seems to be specific for dicots (Reiland et al., 2011). Thus, evolutionary aspects should be taken into account before drawing strict conclusions about the regulation of CEF (see also below).

## The Chloroplast NDH Complex

The chloroplast NDH complex is located in non-appressed thylakoid membranes, and is present in small amounts as compared to major photosynthetic protein complexes. Besides functioning in CEF, NDH is considered to be involved also in chlororespiration (Rumeau et al., 2007). In *Arabidopsis*, the chloroplast NDH complex is composed of more than 30 subunits, which form five subcomplexes: the membrane-embedded subcomplex, the subcomplexes A and B, the electron donor subcomplex and the lumenal subcomplex [for recent reviews, see (Peng et al., 2010; Ifuku et al., 2011)]. The electron donor subcomplex is made of the subunits NdhS, T, U, and V (Yamamoto et al., 2011; Fan et al., 2015), of which the NdhS subunit is responsible for the binding of Fd (Yamamoto et al., 2011; Yamamoto and Shikanai, 2013). The subcomplexes B and the lumenal subcomplex are absent from cyanobacteria. Furthermore, also a liverworth *Marchantia polymorpha* lacks the lumenal subcomplex (Ueda et al., 2012). In angiosperms, the NDH complex further forms supercomplexes with PSI, the minor LHCI proteins Lhac5 and Lhca6 functioning as linkers (Peng et al., 2008, 2009; Peng and Shikanai, 2011; Kouril et al., 2014), whereas in cyanobacteria and *Marchantia*, NDH exists as a single complex. Intriguingly, gymnosperms and *Chlamydomonas* lack the chloroplast NDH complex (Wakasugi et al., 1994; Maul et al., 2002), however, in *Chlamydomonas*, a type-II NDH (NDH-2) has been shown to function in the non-photochemical PQ reduction (Mus et al., 2005; Jans et al., 2008; Desplats et al., 2009).

While the NDH complex plays a crucial role for carbon fixation in the bundle sheath cells of the C4 plants (Majeran et al., 2008), the physiological role of the NDH complex in mature C3 plants has remained largely uncharacterized. However, existence and maintenance of such a massive complex with both nuclear and plastid encoded subunits and a complex assembly pathway (Peng et al., 2009) concomitantly suggest that the NDH complex must bear a crucial role for plant performance. Particularly the drastic phenotype of the *pgr5 crr-2* double mutant even under optimal conditions (Munekage et al., 2004) indicates that the NDH-mediated CEF bears a compensatory role in the *pgr5* mutant background. The exact molecular mechanism for this still remains elusive, particularly as no increase in the level of the NDH subunits has been reported for the *pgr5* mutant (Munekage et al., 2004; DalCorso et al., 2008; Suorsa et al., 2012). However, it has been suggested that NDH might act as a proton pump (Shikanai, 2014), similar to mitochondrial complex I (Baradaran et al., 2013). The role for NDH in proton-pumping and lumen acidification would explain the severe phenotype of *pgr5 crr-2* double mutant. However, experimental evidence demonstrating such a function for NDH is still missing.

The present lack of physiological knowledge on the role of NDH complex likely stems from the fact that majority of the studies concerning the NDH complex have been focused on identification and characterization of the novel subunits and assembly factors, rather than in addressing its functional role under varying environmental and/or developmental conditions. It is highly likely that during the forthcoming years, our knowledge on the physiological role of NDH will be markedly broadened. Indeed, the rice *ndh* mutants were recently shown to exhibit disturbed electron transfer parameters as well as reduced growth and yield particularly under low light conditions, highlighting the physiological significance of the NDH complex under non-optimal growth conditions (Yamori et al., 2015).

The involvement of NDH also in chloroplast redox regulation is already well-documented, and it is conceivable that the NDH-dependent CEF has a role in alleviating oxidative stress under various challenging conditions, such as under drought, extreme temperatures or during early developmental phases (see below). It has been shown that treatment of barley leaves with H_2_O_2_ increased the expression of plastid-encoded NDH genes and subunits (Casano et al., 2001). It was also recently demonstrated that increased levels of H_2_O_2_, occurring either after infiltration of the WT *Arabidopsis* leaves or in mutants producing elevated levels of H_2_O_2_, triggered specifically the NDH-dependent CEF (Strand et al., 2015). In agreement with these observations, *Arabidopsis ndh* mutants showed enhanced levels of foliar H_2_O_2_ upon transfer of plants from darkness to light (Sirpio et al., 2009). On the other hand, *Arabidopsis* mutants with strongly lowered production of glutathione or thylakoid-bound ascorbate peroxidase have been shown to downregulate the expression of several nuclear genes encoding the NDH subunits or assembly factors (Queval and Foyer, 2012).

*Arabidopsis ndh* mutants have been demonstrated to exhibit increased resistance against fungal pathogen (Garcia-Andrade et al., 2013). *Vice versa*, inoculation of the WT plants with fungal pathogens or chitosan, a pathogen-associated molecular pattern that triggers immune responses, resulted in rapid and specific decline in the content of the NDH complex subunit NdhI. More specifically, pathogen exposure was shown to include modulated editing of the chloroplast-encoded NDH transcripts (Garcia-Andrade et al., 2013). The chloroplast NDH complex thus appears to be involved in plant immunity regulation. Intriguingly, an extrinsic PSII oxygen evolving complex protein PsbQ has been shown to be a specific target for pathogen attack (Rodriguez-Herva et al., 2012), and the lumenal subcomplex of NDH contains proteins homologous to PsbQ (Suorsa et al., 2010; Yabuta et al., 2010). However, it still remains to be elucidated whether some subunits or subcomplex(es) of NDH bear more important role in immune responses than others.

## The Role of PGR5 in Acclimation to Fluctuating Light Intensities

Involvement of the PGR5 protein in acclimation to high light has been demonstrated already upon initial characterization of the protein, as the *pgr5* mutants were found to be more susceptible to high light intensities as compared to WT (Munekage et al., 2002). The *pgr5* mutant is incapable of the induction of the NPQ; furthermore, and in contrast to the *npq4* mutant (Grieco et al., 2012; Tikkanen et al., 2015), *pgr5* cannot oxidize its P700 under high light (Munekage et al., 2002). Indeed, high light intensities lead to preferential damage of PSI in *pgr5* plants (Munekage et al., 2002), confirming a crucial role of the PGR5 protein in photoprotection of PSI under high light. In line with its susceptibility for high light, it has been reported that the *pgr5* mutant shows stunted growth under high light intensities, a defect alleviated by increased CO2 concentrations (Munekage et al., 2008).

In any case, it is remarkable that the *pgr5* mutant is capable of growing under constant high light, since growth under fluctuating light, in which low light intensity of 50 μmol photons m^-2^ s^-1^ becomes repeatedly interrupted with peaks of moderately high light (500 μmol photons m^-2^ s^-1^), resulted in lethal phenotype of the *pgr5* mutant (Tikkanen et al., 2010; Suorsa et al., 2012). In order to successfully acclimate for fluctuating light intensity, plants must acquire rapid acclimation shifts between an intense light harvesting mode (low light phase) and a quenching mode (high light phase). Consequently, long-term acclimation strategies applied for growth under constant high light, such as leaf pigmentation, morphological, and ultrastructural changes and constantly enhanced NPQ (Li et al., 2009), cannot be utilized for acclimation for the high light phases of fluctuating light, as they would severely compromise efficient light harvesting under low light phases. Therefore, acclimation to fluctuating light requires specific acclimation mechanisms, of which the so called “photosynthetic control,” i.e., strong down-regulation of electron flow to PSI, bears the most crucial role (for review, Suorsa et al., 2013). Limitation of electron flow to PSI enables its oxidation upon high light phases of the fluctuating light, thus safeguarding the function and stability of PSI. Indeed, *Arabidopsis pgr5* mutants exhibit severe photoinhibition of PSI under fluctuating light (Suorsa et al., 2012; Kono et al., 2014). Furthermore, the rate of CEF has been shown to increase during photosynthetic induction (Fan et al., 2007), which likely prevents PSI acceptor site limitation when the Calvin-Banson-Bassham cycle is not yet properly optimized. The *pgr5* mutant under fluctuating light might thus suffer also due to defects in re-routing the electrons upon photosynthetic induction. However, it is not yet known whether the role of photosynthetic induction is as crucial upon shift from low light to high light, as it is known to be upon shift of plants from darkness to light. Noteworthy, the *Arabidopsis ndh* mutants do not show any phenotype under the fluctuating light conditions described above, indicating the NDH complex does not bear a crucial role for acclimation to light conditions in which light intensity fluctuates between low and moderately high light (Suorsa et al., 2012).

## Involvement of CEF Upon Drought and Extreme Temperatures

Drought stress with subsequent lack of CO_2_ for carbon fixation due to stomatal closure is one of the well-characterized environmental conditions triggering CEF (Golding and Johnson, 2003; Golding et al., 2004; Rumeau et al., 2007; Munekage et al., 2008; Kohzuma et al., 2009; Johnson, 2011). *Arabidopsis* plants overexpressing PGR5 have been shown to exhibit enhanced tolerance to drought stress (Long et al., 2008). Furthermore, drought-stressed *Arabidopsis* plants upregulated the expression of the *PGR5, PGRL1A*, and *PGRL1B* genes with concomitant accumulation of the PGR5 and PGRL1 proteins, whereas both the transcript and the protein levels of the NDH subunit NdhH remained stable (Lehtimaki et al., 2010). On the other hand, under low air humidity but normal watering, the tobacco *ndhb* mutant has been shown to exhibit compromised growth as compared to WT plants (Horvath et al., 2000). Additionally, tobacco *ndhb* mutants were shown to upregulate the PGR5–PGRL1-dependent CEF under drought stress (Munne-Bosch et al., 2005), indicting compensatory roles for the two CEF routes.

It is pivotal to keep in mind that due to evolutionary adaptation to a variety of growth habitats, physiological responses to environmental stress conditions are highly species-dependent, and results obtained with one species cannot always be generalized to others. For instance, in comparison to upregulated levels of PGR5 upon drought stress in *Arabidopsis* (Long et al., 2008; Lehtimaki et al., 2010), *Rosa meillandina*, which is very tolerant against high temperatures and high light as long as there is no shortage of water, showed increased contents of PGR5 as a response to heat and light (Paredes and Quiles, 2013). However, combination of heat, high light intensity and drought stress induced decreased levels of PGR5 with simultaneous upregulation of the NDH complex and plastid terminal oxidase (PTOX), strongly indicating a role for NDH in chlororespiration in *R. meillandina* under those conditions (Paredes and Quiles, 2013).

**FIGURE 1:**
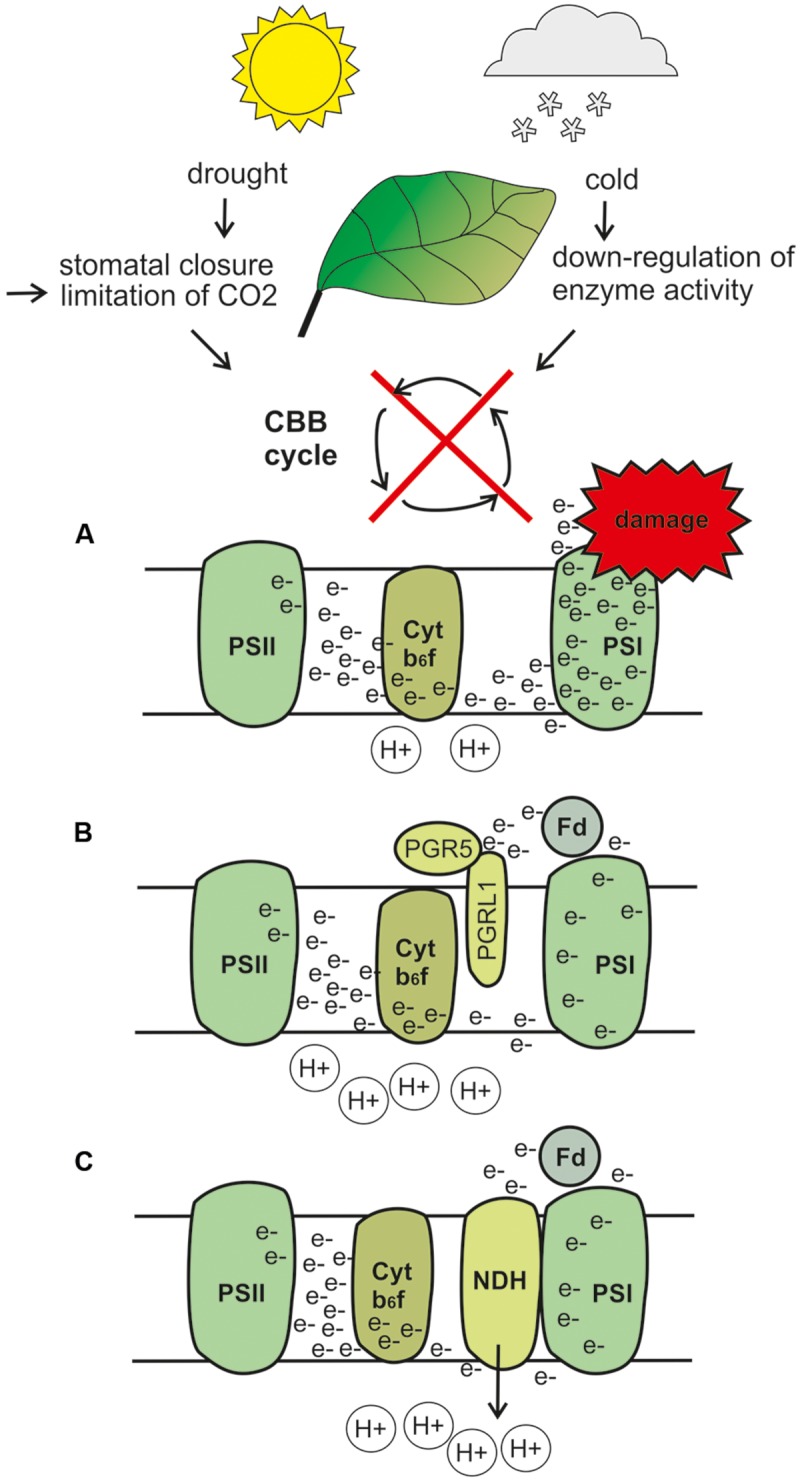
**Hypothetical model describing upregulation of cyclic electron flow (CEF) under drought and cold stress.** Drought stress induces stomatal closure and subsequent limitation in CO_2_ levels, whereas cold stress slows down the enzyme activity. Both conditions result in downregulation of the Calvin-Benson-Bassham cycle. **(A)** A hypothetical situation in which CEF would not function, and which would thus end up in severe over-reduction of the ETC and stroma, and finally to photodamage. **(B)** When the PGR5 and PGRL1 proteins receive electrons from ferredoxin (Fd) and trigger lumen acidification, electron flow toward PSI becomes, thus preventing ETC and stromal components from over-reduction and photodamage. In addition, lumen acidification leads to induction of non-photochemical quenching (NPQ; not drawn to the figure). **(C)** The NDH complex receives electrons from Fd, thus functioning as a safety valve for the excess of electrons. Furthermore, NDH likely functions as a proton pump, which participates in lumen acidification. Note that under most natural conditions, drought and cold likely induce upregulation of both CEF routes to some degree, but the preferential route depends on plant species. The illustrations describe only the hypothetical, not the actual position of the proteins and protein complexes. CBB, Calvin-Benson-Bassham cycle; PSI, photosystem; Fd, ferredoxin; e-, electron; H+, proton.

Similar to drought stress, also cold stress causes lowered carbon fixation, which in turn results in an excessive amount of reducing equivalents and thus imbalanced stromal redox state (**Figure 1**). Cold stress in combination with light illumination threatens particularly PSI (Sonoike and Terashima, 1994; Sonoike et al., 1995; Tjus et al., 1998; Kudoh and Sonoike, 2002), and similar to situation during fluctuating light, CEF plays an important role in protection of PSI also under low-temperature-caused stress. For example, treatment of spinach leaves with low temperatures has been shown to enhance CEF (Kou et al., 2013). In addition, a 3-day-treatment of maize plants with lowered temperature induced upregulation of particularly the PGR-mediated CEF (Savitch et al., 2011). In line with this, cold-acclimated *Arabidopsis* plants showed upregulation of PGR5–PGRL1-dependent CEF, while NDH complex abundancies rather decreased upon cold acclimation (Ivanov et al., 2012). On the other hand, rice mutants lacking the NDH complex showed a growth defect as a response to lowered temperatures (Yamori et al., 2011). Furthermore, the tobacco *ndhb* mutants exposed to a combination of low temperature and low light intensity showed disturbed regulation of electron transfer chain (ETC) as compared to WT (Li et al., 2004). It seems likely that the responses against cold stress in chilling-sensitive plants differ from those of tolerant species, which again highlights the broad variety in CEF responses in different species. Enhanced CEF has also been suggested to be involved in drastic modulations of ETC occurring in conifer needles during winter (Oquist and Huner, 2003), yet experimental evidence is still needed to verify these hypothesis.

Root temperature has been shown to bear particular significance for the temperature responses in terms of CEF regulation. Visible damage and complete blockage of both LEF and CEF occurred in rice when only stem, but not root temperature was lowered (Suzuki et al., 2011). Furthermore, this condition has been shown to upregulate both NDH complex and the plastid terminal oxidase contents in a tropical plant *Spathiphyllum wallisii*, whereas upregulation of the PGR5–PGRL1 – dependent CEF as well as that of the PGR5 levels were observed either when entire plant was chilled (Segura and Quiles, 2015), or when roots were chilled but stems heated (Soto et al., 2014).

## CEF Plays an Important Role under Early Developmental Phases

Cyclic electron flow seems to play a crucial role under the early developmental stages. A recent comprehensive study on photosynthetic features of *Arabidopsis* seeds demonstrated that the light which the embryo chloroplasts receive is enriched in far red region of the spectrum, which preferentially excites PSI (Allorent et al., 2015). Consequently, green seeds were found to possess an increased CEF as compared to leaves, and the seed germination rates positively correlated with CEF activity (Allorent et al., 2015). Notably, even though in C3-leaves the proportion of the PGR5–PGRL1-dependent CEF is higher than that of the NDH-dependent CEF, in seeds the latter one plays a more prominent role (Allorent et al., 2015). The importance of the NDH-dependent CEF route under early developmental phases is in line with earlier reports showing that the NDH-complex subunits are present and assembled as subcomplexes already in etioplasts (Kanervo et al., 2008), and the final assembly of the PSI-NDH supercomplex rapidly takes place upon exposure of etioplasts into light (Kanervo et al., 2008; Peng et al., 2009).

The enhanced CEF under early developmental stages highlights the proposed role of CEF in photoprotection of PSI. We have previously shown that photoprotection of PSI by the PGR5 protein under fluctuating light plays its most crucial role upon early developmental stage (Suorsa et al., 2012, 2013). Indeed, the synthesis of PSI is most active in young leaves, which causes strong decline in the contents of the PSI assembly factors upon leaf maturation (Krech et al., 2012; Liu et al., 2012). This corroborates with the idea of PSI being vulnerable and thus in the need of intensive protoprotection particularly under early developmental states.

The role of CEF during the opposite phase of leaf development, i.e., under senescence, still remains elusive. Similar to early developmental phases, leaf senescence involves oxidative stress. Early phases of the senescence are characterized by upregulation of the antioxidant machinery, which allows controlled remobilization and recycling of nutrients and photoassimilates to other parts of the plants (Juvany et al., 2013). Upon later senescence, declining antioxidant network induces massive oxidative stress, which leads to damage and ultimately to death. Intriguingly, it has been shown that tobacco plants lacking the chloroplast-encoded NDH subunit NdhF, and thus containing only residual amounts of the NDH complex, exhibited delayed senescence under optimal greenhouse conditions (Zapata et al., 2005, 2007). Furthermore, the tobacco Δ*ndhF* mutants showed increased fitness, likely due to delayed senescence (Zapata et al., 2007). However, the exact molecular mechanism(s) behind the delayed senescence in the tobacco Δ*ndhF* mutant remain to be characterized, neither it is known whether the effect can be generalized to other long-lived plants.

## Concluding Remarks

During the past few years, knowledge on the protein subunits and assembly factors involved in the two main routes for CEF has been substantially increasing. The available information about molecular mechanisms behind CEF is now enabling more intense research focus being directed toward physiological significance of CEF. There is already compelling evidence about the significance of particularly the NDH-dependent CEF for the energy metabolism in developing embryos. Both routes of CEF also seem to respond to stresses induced by drought or coldness. Furthermore, the PGR5 protein has been shown to have a crucial role in acclimation to fluctuating light conditions. However, results concerning CEF that have been acquired with one species cannot be necessarily generalized to cover all species. Thus, future studies with a wide range of evolutionary divergent species are still needed in order to obtain a more comprehensive view on the impact of CEF on plant development and environmental acclimation.

## Conflict of Interest Statement

The author declares that the research was conducted in the absence of any commercial or financial relationships that could be construed as a potential conflict of interest.
